# Fluorescence imaging in vivo visualizes delayed gastric emptying of liquid enteral nutrition containing pectin

**DOI:** 10.1186/1471-230X-14-168

**Published:** 2014-09-27

**Authors:** Ippei Yamaoka, Takeshi Kikuchi, Naoyuki Endo, Goro Ebisu

**Affiliations:** Medical Foods Research Institute, OS-1 Division, Otsuka Pharmaceutical Factory, Inc, 115 Kuguhara, Tateiwa, Muya-cho, Naruto, Tokushima, 772-8601 Japan; Naruto Research Institute, R & D Center, Otsuka Pharmaceutical Factory, Inc, 115 Kuguhara, Tateiwa, Muya-cho, Naruto, Tokushima, 772-8601 Japan

**Keywords:** Mouse, IVIS, Semi-solid, Gelation, Gastrosense

## Abstract

**Background:**

Semi-solidification by gelation or increased viscosity could slow the influx of liquid enteral nutrition (EN) into the small intestine. A liquid EN formula containing pectin that gels under acidic conditions such as those found in the stomach has been developed. A new near-infrared fluorescent imaging reagent was used to non-invasively acquire real time images of gastric emptying in a murine model in vivo. We postulated that the EN formula delays gastric emptying and tested this hypothesis using imaging in vivo.

**Methods:**

Male BALB/c mice were given an oral bolus injection of a liquid EN containing the fluorescence reagent GastroSense750 with or without pectin. The EN in the stomach was visualized in vivo at various intervals using a non-invasive live imaging system and fluorescent signals were monitored from the stomach, which was removed at 60 min after EN ingestion.

**Results:**

The fluorescence intensity of signals in stomachs in vivo and in resected stomachs was lower and attenuated over time in mice given EN without, than with pectin.

**Conclusions:**

Adding a gelling agent such as pectin delayed the transit of liquid EN from the stomach. Fluorescence imaging can visualize the delayed gastric emptying of EN containing pectin.

## Background

An increase in the caloric content of a liquid meal causes delayed gastric emptying of the meal [1]. However, ingestion beyond an acceptable volume, which is common in patients who undergo gastric and esophageal surgery, fundoplication, or bariatric surgery, leads to the rapid infusion of the liquid meal into the small intestine [2–4]. The accelerated liquid meal transition through the small intestine results in gastrointestinal symptoms (abdominal pain, diarrhea, borborygmi, nausea and bloating) and vasomotor symptoms (fatigue, facial flushing, hypotension and perspiration) that are classified as an early symptom of dumping syndrome. Reducing the infusion speed using a syringe pump is a simple and effective method to avoiding such risk [5], but the cost is high and the procedure is awkward in the clinical setting. Increasing the viscosity of a meal with dietary fiber is another approach to relieving the symptoms of dumping syndrome by slowing gastric evacuation [6].

Pectin is a water-soluble viscous polysaccharide with low- or high-methoxyl formulations, depending on the content of methoxy-modified galacturonic acid. The mechanism for low methoxyl-pectin gelation is via the binding of calcium ions to pectin homogalacturonic regions [7]. We developed a liquid enteral nutrition (EN) product containing low-methoxy pectin, in which the calcium changes from an electrovalent state to an ionized state under acidic conditions and thus promotes pectin gelation. The liquid EN formulation would also slow its own gastric transition into the small intestine because highly viscous liquid meals containing pectin delay gastric emptying [8, 9]. In contrast to these findings, another study has found that highly viscous liquid EN with pectin accelerates gastric emptying [10].

Diagnostic MRI and scintigraphy, which can directly visualize EN distribution in the stomach, are a powerful tool with which to assess gastric emptying in humans. Moreover, rapid MRI can visualize gastric emptying and duodenal motility simultaneously in humans [11]. Diagnostic MRI and scintigraphy are proposed to be useful in intact animals [12–14]. However, the high cost and/or technical hurdle to high speed of gastric function and smaller size of animals would deter general application of these techniques for routine screening in rodents. Gastric emptying has historically been visualized using residual gastric and duodermal beads, dyes, radioactive labelling or phenol red [15, 16]. However, these methods require animal sacrifice and several animals for every point of interest. Continuous ^13^C-breath tests enable non-invasive assessment of the gastric EN transition [17], but the techniques are indirect.

A novel near-infrared fluorescent imaging reagent was developed to non-invasively acquire real time images of gastric emptying from a murine model in vivo. Thus, we reasoned that the technique is applicable for the elucidation of gastric emptying of EN and could reveal the impact of pectin on gastric emptying by direct imaging of gastric EN residue. Here, we compared the fluorescence intensity determined at various intervals from the surface of the stomach of the same individuals that received an oral bolus of liquid EN containing the fluorescence reagent GastroSense750 (Perkin-Elmer, Waltham, MA, USA) and with or without pectin. We tested the hypothesis that fluorescence imaging could visualize delayed gastric emptying of EN caused by adding pectin to enteral nutrition.

## Methods

**Figure 1:**
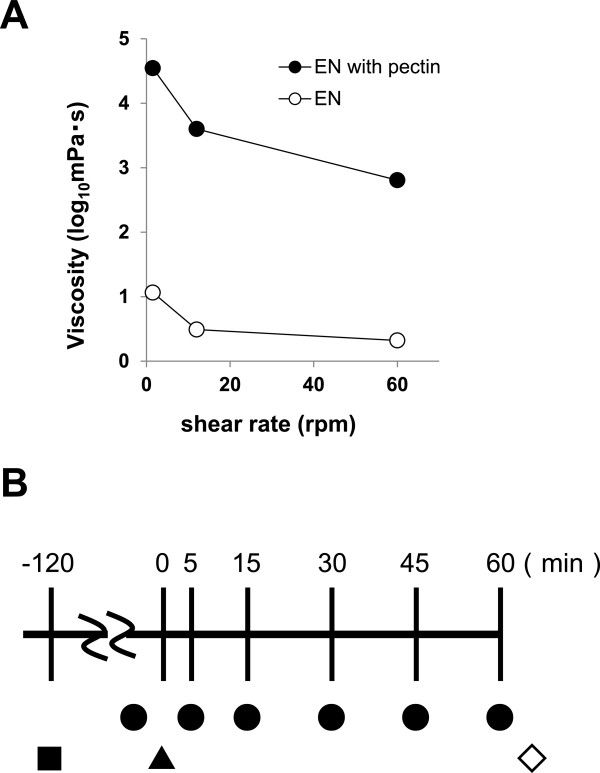
**Viscosity of enteral nutrition mixed with artificial gastric juice and schema of experimental design. A**. Effect of shear rate on viscosity measurements of EN with (filled circles) and without (unfilled circles) pectin mixed with equal amount of artificial gastric juice. **B**. Schema of experimental design. Mice fasted for 24 hrs were injected with 10 μL/g body weight of enteral nutrition (0 min; ▲) 2 h after withdrawing drinking water (■). Gastric imaging proceeded 5, 15, 30, 45, 60 minutes after injection under isoflurane anesthesia (●). Isolated stomachs were assessed thereafter (◇).

This study examined two liquid EN formulations that differed in terms of viscosity after acidification and the presence or absence of pectin (Table 1, Figure 1A). Adding pectin increased the physical viscosity of the formulation as ionized calcium concentrations increased at low pH. Five minutes after stirring 50 mL of artificial gastric juice comprising 12 M HCl (7 mL/L) and NaCl (2 g/L) into 50 mL of each formulation, the viscosity of the EN formulations after the acidification was measured at 1.5, 12, and 60 rpm at 25°C using an TVB-22 L Brookfield type viscometer (Figure 1A, Toki Sangyo Co. Ltd., Tokyo, Japan).

**Table 1:** **Energy, macronutrient composition, dietary fiber and viscosity of liquid enteral nutrition formula with or without pectin**

	EN	EN with pectin
Energy, kcal/100 mL	78.6	80
Protein; Carbohydrate; Fat, g/100 kcal	4.0; 15.9; 2.2	4; 16.8; 2.2
Total Dietary Fiber, g/100 kcal	0.5	1.4
- Pectin^1^, g/100 kcal	0	0.9
Viscosity of EN, mPas ∙ s^2^	4.9	9.0

EN, Enteral nutrition. ^1^Calculated pectin calories, 2 kcal/g. ^2^Measured at 12 rpm at 25 degrees C using Brookfield Viscometer.

Sixteen 5-week-old male BALB/c CrSlc mice (Japan SLC, Inc. Hamamatsu, Shizuoka, Japan) weighing 18–23 g were housed in cages contained bedding under constant 55% ± 15% humidity at a room temperature of 23 ± 3°C under a 12:12 h light-dark cycle (lights on, 07:00 h–19:00 h). Vendor health reports indicated that the mice were free of known viral, bacterial and parasitic pathogen. The Committee for the Care and Use of Laboratory Animals at Otsuka Pharmaceutical Factory Inc. approved the experimental procedures associated with this study. Mice were acclimatized for at least 4 days and allowed to free access to water and a commercial maintenance diet (CRF1, Oriental Yeast Co., Ltd., Tokyo, Japan). Mice with the same average body weight were then assigned to two groups of 8 mice each: A. Mice given EN without pectin; B. Mice given EN with pectin, at least two weeks after feeding ad libitum with the Ivid#1 diet (Oriental Yeast Co., Ltd.) that is specific for imaging in vivo and free access to water. A preliminary study revealed that to achieve power = 0.08 and alpha =0.05 to detect mean effect size of pectin would require 5 animals per group. All the following experiments were conducted in the light phase. The experiment was repeated configured groups of mice are used both (e.g. sequence A-B then B-A) and data were pooled. Experimental procedure was shown in Figure 1B. The mice were fasted for 24 h without water for the last 2 h to eliminate any impact of extant gastric nutrients and water and thus minimize variations in gastric H^+^ concentrations among the mice. Subsequently, the amount of fluorescence at the body surface of the mice before ingesting the test EN solution was monitored under 2.5% isoflurane anesthesia and then the mice were placed on a heating pad to recover. The entire procedure from induction to arousal required about 3 min. Thereafter, the mice were gavaged with 10 μL of liquid EN/g of body weight containing 1.25 pmol GastroSense™750 fluorescent imaging agent (Perkin Elmer Inc.). We compared a liquid EN (Hine® E-gel, Otsuka Pharmaceutical Factory, Inc., Tokushima, Japan) containing 0.88 g/100 kcal low-methoxyl pectin (CP Kelco, Lille Skensved, Denmark) and a similar EN without pectin. The mice were then allowed to move freely in plastic cages except when placed in an apparatus under isoflurane anesthesia for imaging at intervals of 5, 15, 30, 45 and 60 min. After 60 min of imaging, the mice were sacrificed under continued isoflurane anesthesia and the gastrointestinal tracts were removed. The distribution of GastroSense™750 fluorescence in the stomach in vivo and after resection was monitored using the IVIS® Spectrum live animal imaging system (Perkin Elmer Inc.) with excitation and emission at 745 and 800 nm, respectively. Regions of interest (ROI) were placed over the location corresponding to the stomach on the skin surface of the mice to determine total radiant efficiency. Bioluminescence was quantified using IVIS® imaging software (Perkin Elmer), which generated the total flux of radiance (photons/second emitted from the surface) in each pixel, summed over the ROI area per cm^2^ of tissue. Average photon radiance is displayed, which is the sum of the radiance from each pixel inside the ROI/number of pixels. The primary outcome measures were the impact of pectin on the transition of gastric fluorescence emitted by EN residues in vivo.

All values for each group are presented as means ± SD. Differences between groups were analyzed by two-way ANOVA for repeated measures followed by Student’s t-test where appropriate. Statistical significance was set at p < 0.05.

## Results

**Figure 2:**
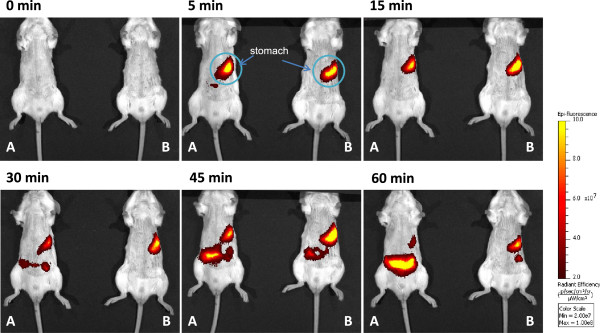
**Representative ventral images of fluorescence emission of gastric contents.** Mice received a bolus of 10 μL/g body weight of liquid EN (Hine® E-gel) containing 1.25 pmol of GastroSense™750 fluorescence imaging agent without **(A)** or with **(B)** pectin. Representative ventral images of anesthetized mice are shown at baseline and at 5, 15, 30, 45, and 60 min later to monitor gastric EN. All images were acquired using the same pseudocolor scale of radiance to show relative changes in bioluminescence emission over time.

**Figure 3:**
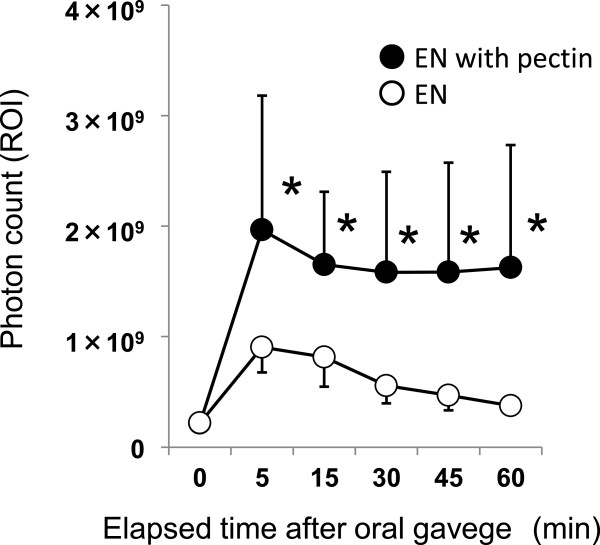
**Gastric residue of enteral nutrition in mice.** Gastric fluorescence is represented on Y-axis as measure of gastric residue of enteral nutrition. Values from mice weighing 22–25 g (n = 8 per group) without (unfilled circles) and with (filled circles) pectin over time are shown as means ± SD. *p < 0.05 (Student’s t test).

**Figure 4:**
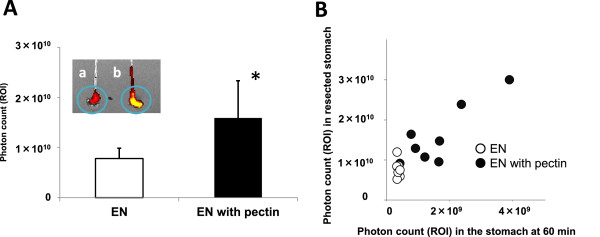
**Fluorescence emission in enteral nutrition residues from resected stomachs of mice at 60 min after ingestion. A**. Representative images of fluorescence values derived from gastric contents of mice that were given liquid EN bolus containing fluorescent imaging agent without (a) or with (b) pectin. Fluorescence emission on Y-axis as indicates gastric residues of enteral nutrition. Values from mice (n = 8 per group) without (unfilled bars) and with (filled bars) pectin are shown as means ± SD. *p < 0.05 (Student’s t test). **B**. Regression analysis of fluorescence emitted by enteral nutrition residues with (filled circles) and without (unfilled circles) pectin in resected stomachs vs. stomachs imaged at 60 min after ingestion in vivo.

The EN without pectin was found in the mouse stomach at 5 and 15 min after gavage. The signal became attenuated thereafter and fluorescence emission became evident around the small intestine and intestinal cecum over time. The EN containing pectin remained in the stomach and intestinal fluorescence emission remained weak throughout the study (Figure 2). The fluorescence intensity in the stomach of mice given EN with pectin was significantly higher at 5 min after ingestion and remained elevated throughout the study (Figure 3). The fluorescence intensity in the resected stomach was also significantly higher in the mice given EN with pectin (Figure 4A). Figure 4B shows the correlation between fluorescence emission in EN residues with and without pectin between the resected stomach and the stomach imaged in vivo at 60 min after ingestion. Higher intensity in the resected stomach reflected that in the stomach in vivo (r = 0.90, p < 0.05; n = 16).

## Discussion

The present study showed that visualizing a fluorescence probe dissolved in liquid EN in the mouse stomach at intervals is useful for detecting changes in gastric emptying caused by the physical properties of the EN. Furthermore, gastric emptying was delayed in mice that received liquid EN with pectin than in those that did not.

Pectin slowed gastric emptying 5 min after ingestion. The ^13^C-breath test in mice has shown that >100% of the dose/h of ^13^CO_2_ appears from 5 min after ingesting a liquid meal bolus [17]. The average weight of the small intestinal contents of rats after forced feeding with a liquid diet reaches a maximum after 20 min [18]. These facts support the present finding that fluorescence imaging visualized the immediate transition of liquid EN without pectin into the small intestine after ingestion. Moreover, this finding suggested that pectin changes gastric fluidity quite promptly. Dietary volume, viscosity, osmotic pressure, chemical composition and pH are factors involved in the regulation of gastric emptying [1]. Since pectin affects only viscosity [19], the viscosity of EN with pectin should change in the stomach from quite an early phase. That EN with pectin rapidly formed a gel after mixing with artificial gastric fluid supported the present findings. Furthermore, although the viscosity of the material responded inversely to higher shear rates (shearing thinning), the viscosity of EN containing pectin after acidification was greater than that of EN without pectin at all shear rates examined (Figure 1A). The assumption that the higher viscosity of EN with pectin in the stomach has inhibitory effects on gastric emptying seems reasonable; however, accurate shear rates in the rumen are unknown. The fluorescence intensity of the stomach became attenuated and persisted in mice given EN without and with pectin, respectively. Fluorescence intensity in the resected stomachs from mice receiving EN with pectin also persisted, indicating that pectin delayed gastric emptying throughout the experimental period. Adding pectin retards the gastric emptying of not only liquid, but also of solid food [8]. In addition to liquid gelation, pectin might also induce delayed gastric emptying via other factors such as duodenal feedback regulation. Soluble and viscous fibers such as pectin slow digestion and nutrient absorption and prolong the presence of nutrients in the critical region. Therefore, nutrients that lag in the duoderm continue to elicit factors involved in the feedback mechanism. If duodenal feedback is regulated due to initial volume-dependent outflow in pectin-free controls, a mechanism evoked by increased viscosity would predominate in controlling gastric emptying.

Consensus has not been reached regarding the role of pectin in gastric emptying among studies suggesting that liquid meals with pectin lead to delayed emptying responses [8, 9], a delay during the last 20% of a meal [20], or an accelerated response [10]. The dose of pectin added to a liquid meal might alter the response of the gastric emptying. Sandhu et al. found that medium or higher doses of pectin delayed gastric emptying, whereas lower doses did not [8]. Adding 5.2 g of pectin per 500 mL and 450 kcal of energy to liquid EN meal delayed gastric emptying. The pectin dose administered in the present study (0.9 g/100 kcal/125 mL of EN) approximates the study of Sanaka et al. [9]. In contrast, a much lower dose of pectin (2 g/654.5 kcal diet) has delayed emptying of the last 20% of a meal [21] and a lower dose of pectin (1.4 g followed by 400 mL EN) increases gastric emptying rates [10]. Because a higher pectin concentration renders EN more viscous, differences in the amounts of added pectin result in EN with different levels of viscosity, which leads to differences in the impact of pectin on gastric emptying among studies. Furthermore, differences in the fat content (2.2 g/100 kcal in our study) of the EN used as controls might explain the discrepant findings, because increased lipid content slows gastric emptying [21]. In fact, although adding pectin to EN containing 1.3 g/100 kcal fat delayed gastric emptying [9], pectin ingestion before administering the EN containing more fat (3.7 g/100 kcal) enhanced it [10].

Different methods of measuring gastric emptying might also contribute to differences in the effects of pectin on gastric emptying. We non-invasively and directly acquired real-time images of gastric emptying in a murine model in vivo using a novel near-infrared fluorescence imaging reagent. Since polysaccharides such as pectin affect not only the gastric emptying of ingested materials but also intestinal transition and absorption [22, 23], the effects of pectin on gastric empting cannot be measured using the ^13^C breath test without considering the impact on the intestine. Consequently, we directly visualized ingested substances and their attenuation in the stomach.

This study has some limitations. The mice were anesthetized with isoflurane at intervals to investigate gastric imaging although mice are aroused at times other than these periods. Such anesthesia limited the gastric transition of EN, as common anesthetics delay gastric emptying in animals [24]. Second, slight positional differences of EN in the mouse stomach altered signal intensities regardless of how well we spread-eagled the mice on their backs for each observation. As far as we know, the depth of the target EN from the surface and the physiological condition encircling illuminant define the intensities. Nonetheless, this experimental approach to measure gastric emptying of EN allows for reductions in lethal and invasive animal experiments.

## Conclusion

We showed that adding a gelling agent such as pectin slows the removal of liquid EN from the stomach and that fluorescence imaging can visualize delayed gastric emptying caused by adding pectin to EN.

## Abbreviation

EN: Enteral nutrition.
